# Investigation of Feeding Problems and Their Associated Factors in Children with Developmental Disabilities in Saudi Arabia

**DOI:** 10.3390/nu18020356

**Published:** 2026-01-22

**Authors:** Walaa Abdullah Mumena, Sara Zaher, Maha Althowebi, Manar Alharbi, Reuof Alharbi, Maram Aloufi, Najlaa Alqurashi, Rana Qadhi, Sawsan Faqeeh, Arwa Alnezari, Ghadi A. Aljohani, Hebah Alawi Kutbi

**Affiliations:** 1 Clinical Nutrition Department, College of Applied Medical Sciences, Taibah University, Madinah 42353, Saudi Arabia; 2 Health and Life Research Center, Taibah University, Madinah 42353, Saudi Arabia; 3 Department of Clinical Nutrition, Faculty of Applied Medical Sciences, King Abdulaziz University, Jeddah 21589, Saudi Arabia

**Keywords:** feeding problems, developmental disabilities, children, diet, Saudi Arabia

## Abstract

Background/Objectives: Children with developmental disabilities (DD) may experience feeding problems that increase their risk of malnourishment. However, data concerning factors linked to feeding problems in children with DD are lacking. The present study aimed to investigate feeding problems and their associated factors in children with DD who are fed orally. This cross-sectional study included data from 160 children with DD aged 2–18 years, recruited from 9 disability centers and schools located in Madinah, Saudi Arabia. Methods: A total of 666 envelopes were distributed randomly to children to take home. Caregivers were asked to provide sociodemographic, health, and nutrition information. Feeding problems were assessed using a validated screening tool for eating/feeding problems (STEP-AR), which included 17 items divided into 5 subdomains (Aspiration risk, Food refusal, Food selectivity, Nutrition behaviors, and Skill). Phone interviews were conducted with caregivers within two weeks of data collection for dietary assessment. Results: The most frequently reported feeding problems involved feeding skills and food selectivity, with 39.3% unable to feed themselves, 33.1% showing overeating behavior, and 31.2% exhibiting pica-like behavior. Chewing difficulties (28.7%), limited food intake (25.6%), and swallowing challenges (21.2%) were moderately reported, while aspiration-related problems were less common. Multiple linear regression analysis revealed significant positive associations between feeding problems and caregiver education level, family income, caregiver’s relationship to the child, and the child’s living arrangement. Dietary intake was not associated with feeding problems. Conclusions: The findings of this study indicate a range of feeding problems and key sociodemographic factors associated with feeding problems in children with DD. These results highlight the need for targeted interventions such as behavioral support and caregiver education to effectively address and manage feeding challenges in children.

## 1. Introduction

Proper nutrition is essential for the growth and development of all children [1]; however, maintaining adequate nutritional status can be particularly challenging in those with developmental disabilities (DD), who often face greater nutritional challenges than their neurotypical peers [2]. While some children with disabilities require enteral nutrition to meet their nutritional needs, a considerable proportion are able to eat orally yet still experience clinically significant, though comparatively less severe, feeding difficulties that may compromise dietary adequacy [3,4]. Children with DD often experience feeding difficulties stemming from a variety of physical, sensory, behavioral, and cognitive impairments. These may include oral-motor dysfunction, food selectivity, gastrointestinal issues, and communication limitations, all of which can result in inadequate dietary intake and consequently poor growth outcomes.

DD comprise a group of conditions that arise during the developmental period and can significantly impair intellectual, motor, language, or social functions [5]. Common types include intellectual disabilities, attention deficit hyperactivity disorder (ADHD), autism spectrum disorder (ASD), learning disabilities, and cerebral palsy [6]. Developmental disabilities can impact children’s health and quality of life, affecting learning, communication, and social interactions [7]. Globally, over 300 million children and young people are estimated to be affected by DD, with risk factors ranging from genetic and perinatal causes to environmental and socioeconomic influences [7].

Children with DD often face challenges that might be experienced because of underlying physical conditions, side effects of medications, or other health issues [8,9,10]. These challenges can contribute to an increased risk of nutrition-related problems, which are common among children with disabilities due to various reasons, such as oral motor difficulties, sensory sensitivities [11], gastrointestinal disorders, or cognitive impairments [12], all of which can lead to food refusal [13], chewing or swallowing difficulties [14], as well as communication difficulties, making it hard for them to express their needs [15]. Consequently, children with DD may face challenges in maintaining a balanced diet, which may cause malnutrition and growth concerns [16,17,18,19,20]. Indeed, the risk of feeding difficulties was found to be higher in children with DD compared to healthy children [21]. Studies exploring factors associated with feeding problems in children with DD in Saudi Arabia are currently lacking. Thus, we aimed in this study to examine feeding difficulties among orally fed children with disabilities who present with clinically relevant yet subthreshold feeding problems that do not warrant artificial nutritional support. The findings of this work will guide targeted interventions to enhance the nutritional status and overall health of children with DD and help identify feeding challenges that may compromise dietary adequacy if unrecognized.

## 2. Materials and Methods

### 2.1. Study Design and Population

This cross-sectional data included orally fed children with DD aged between 2 and 18 years. Children on artificial nutritional support (enteral or parenteral nutrition) were excluded, as their feeding problems differ in nature from oral feeding difficulties. Children with incomplete data were excluded from this study due to different types of feeding problems. The number of children needed for this study was 73 at minimum based on an expected proportion of children with DD of 13%, 0.15 effect size, 95% confidence level, and 90% power. The sample size calculation was performed using G*Power software (version 3.1.9.7).

Ethical approval to conduct this study was obtained from the Ethical Committee of the College of Applied Medical Sciences at Taibah University (certificate number: 2024/177/203 CLN). A signed consent form was obtained from all caregivers before data collection.

### 2.2. Data Collection

Data were collected from six disability centers and three schools between December 2023 and March 2024. Children were selected at random in centers with large numbers of children enrolled and centers/schools with limited numbers of children with DD. All children received envelopes to take home. Children with various types of disabilities, including developmental, physical, behavioral, and sensory impairments, were enrolled at the institutions included in the study.

A total of 666 envelopes were distributed to children to take home, each containing the study objectives, inclusion and exclusion criteria, a parental consent form, and a questionnaire. Only children who met the inclusion criteria described in the questionnaire were expected to return the envelopes. Envelopes were collected within two weeks, and caregivers were later contacted to arrange a phone interview to collect the dietary data of the children. The questionnaire requested caregivers to provide personal data (including contact information) as well as information about their child with DD (diagnosed health condition, feeding problems, and dietary quality).

### 2.3. Sociodemographic and Health-Related Characteristics of the Study Sample

Caregivers of children were requested to provide data concerning their relationship with the child (Mother = 1; Father = 2; Sibling = 3; Other = 4); Caregiver’s age group (20–30 years = 1; 31–40 years = 2; >40 years = 3); Caregiver’s education level (<High school = 1; High school/diploma = 2; Bachelor = 3; Postgraduate degree = 4); Caregiver marital status (Married = 1; Single = 2); Family monthly income in Saudi Riyal (SAR) (<SAR 4000 = 1; SAR 4000–6000 = 2; SAR 6001–10,000 = 3; SAR 10,001–15,000 = 4; >SAR 15,000 = 5); Gender (Boy = 1; Girl = 2); Age Group (1–3 years = 1; 4–8 years = 2; 9–13 years = 3; 14–18 years = 4); Nationality (Saudi = 1; non-Saudi = 2); Order of child (Only child = 1; Oldest child = 2; Middle child = 3; Youngest child = 4); Living arrangement (Live with both parents = 1; Live with mother = 2; Live with father = 3; Live with other family member or in disability center = 4); Type of diagnosed disability (ADHD/ASD/intellectual disability/learning disability/other = 1; Cerebral palsy = 2; Down syndrome = 3); Other Medical Conditions (No = 0; Yes = 1); Untreated dental problem (No = 0; Yes = 1); Medication use (No = 0; Yes = 1); Structured PA (No = 0; Yes = 1); Independency (No = 0; Yes = 1); Sleep time (Adequate sleep = 1; Inadequate sleep = 2); Screen time (1–2 h/d = 1; > 2 h/d = 2); Food allergies (No = 0; Yes = 1); Dietitian visit (No = 0; Yes = 1); Supplement use (No = 0; Yes = 1); Nutritional drink use (No = 0; Yes = 1).

### 2.4. Assessment of Feeding Problems

A validated screening tool for eating/feeding problems (STEP-AR) was used to assess feeding problems in children with DD [22]. The tool contains 17 Likert-type questions divided into five major subdomains regarding feeding problems (Aspiration risk, Food refusal, Food selectivity, Nutrition behaviors, and Skills). There are three responses for each question (never occurs = 0; occurs between 1 and 10 times within the last month = 1; occurs more than ten times within the previous month = 2) and severity (causes no harm = 0; causes minimal harm = 1; causes severe harm or injury = 2). Total feeding difficulties were calculated by measuring the sum of the responses to the STEP-AR questions, which ranged between 1 and 17 [22,23]. The total feeding problem score was calculated by summing the frequency and severity scores across all items, with a maximum possible score of 68, where higher scores indicate greater feeding problem severity.

### 2.5. Assessment of Dietary Quality

Children’s dietary quality was assessed using a short-form food frequency questionnaire (SFFFQ) developed by Cleghorn and colleagues [24], which was previously used in studies conducted in Saudi Arabia [25,26]. It encompasses twenty food items: “Fruits,” “Fruit juice,” “Salad,” “Cooked vegetables,” “Fried potatoes/Chips,” “Beans or legumes,” “Fiber-rich breakfast cereal,” “Whole wheat bread,” “Cheese/Yoghurt,” “Crisps,” “Sweets,” “Ice cream/Cream,” “Soda,” “Beef or lamb,” “Chicken or turkey,” “Processed meats/Meat product,” “Processed chicken/Turkey,” “Fried white fish,” “Whitefish,” and “Oily fish.” The corresponding consumption frequencies for items 1–13 were: “Never or Rarely,” “<1 time per week,” “1 time per week,” “2–3 times per week,” “4–6 time per week,” “1–2 times per day,” “3–4 times per day,” and “≥5 times per day.” The food frequencies for items 14–20 were: “Never or rarely,” “<1 time per week,” “1 time per week,” “2–3 times per week,” “4–6 times per week,” and “≥7 times per week.” The printed SFFFQs were collected from participants and entered in a digital spreadsheet (www.nutritools.org) to calculate diet quality (0–51).

### 2.6. Statistical Analysis

Data analyses were conducted using SPSS (IBM SPSS Statistics for Windows, Version 20). Continued variables are presented as mean ± standard deviation (SD) and median (interquartile range [IQR]), while data of categorical variables are presented as frequency and percentages. Descriptive statistics are reported for demographic characteristics, health-related characteristics, and distribution of responses to STEP-AR. The normality of distribution for feeding problem scores were assessed using the Shapiro–Wilk test, which suggested skewed data. Mann–Whitney U and Kruskal–Wallis tests were used to compare median feeding problems across different groups. Multiple linear regression analysis of feeding problems with sociodemographic variables was conducted, adjusted for disability category. Additionally, multiple linear regression analysis was performed to explore the association with dietary intake in children with DD, adjusted for children’s age, gender, and disability category. All tests were two-tailed, with alpha = 0.05.

## 3. Results

### 3.1. Sample Characteristics

**Figure 1:**
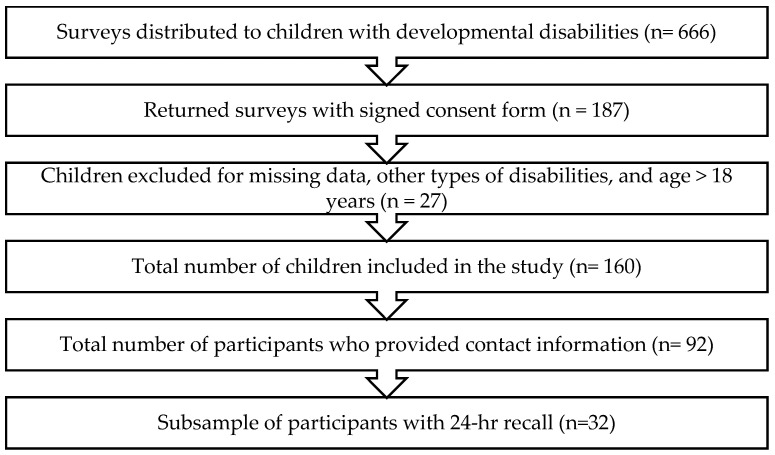
Flowchart of the study sample.

The total number of children included in the final analysis was 160 after excluding 27 children with other types of disabilities, >18 years, and missing data (Figure 1). Most caregivers of children included in this study were mothers (86.9%, *n* = 139), and 47.5% (*n* = 76) of caregivers were aged between 31 and 40 years. Over half of the caregivers reported holding a high school/diploma or less (53.7%, *n* = 86), and 81.9% (*n* = 131) of caregivers were married. Fifty-four percent (*n* = 87) of caregivers reported a family income of ≤SAR 6000 per month. Over half of the children were boys (57.5%, *n* = 92), 45.6% (*n* = 73) of children were between the age of 4 to 8 years, and 89.4% of children were Saudi nationals (*n* = 143). Over one-third (39.4%, *n* = 63) of children were the youngest children in the family, and 83.1% (*n* = 133) of children were living with both parents. Detailed data regarding the demographic characteristics of children are presented in Table 1.

### 3.2. Health-Related Characteristics of the Study Sample

Types of DD among children included in this study were ASD/ADHD/learning disabilities/intellectual disability/other (80.0%, *n* = 128), cerebral palsy (13.8%, *n* = 22), and Down syndrome (6.30%, *n* = 10). Most children (71.9%, *n* = 115) had no other medical conditions. Forty-four percent of children experienced dental issues (*n* = 70) and 19.4% (*n* = 31) were taking medications, of which 46.9% (*n* = 15) were anti-epileptics (Keppra, Tegretol, Levetam, Depakine, Epitam, Topamax, Escitam). One-quarter of caregivers (25.6%, *n* = 41) reported that their children were involved in structured physical activity, while 45.0% were independent (*n* = 72). Thirty-eight percent (*n* = 61) of children had adequate sleep, and 63.8% of children spent ≤2 h on screen (*n* = 102). Only 23.1% (*n* = 37) of children reported visiting a dietitian, while most children were not using any supplements (70.6%, *n* = 113) (Table 2).

### 3.3. Feeding Problems

Limited feeding problems were reported among the study sample, with a mean feeding problem score of 6.23 ± 7.08 and median of 4.00 (1.00–9.75) out of a maximum score of 52. The most commonly reported feeding problems were related to feeding skills and food selectivity. Specifically, 39.3% of children were unable to feed themselves independently. Overeating behavior was observed in 33.1% of children, who continued eating as long as food was available. Additionally, 31.2% of the sample exhibited pica-like behavior, attempting to eat non-food items. Chewing difficulties (28.7%), limited food intake (25.6%), and swallowing challenges (21.2%) were reported at moderate levels. By contrast, aspiration-related problems such as regurgitation (14.3%), choking (13.7%), and vomiting (7.5%) were less frequent. A detailed description of responses to items included in the STEP-AR is provided in Table 3.

### 3.4. Association Between Feeding Problems and Sociodemographic Characteristics of the Study Sample

Non-parametric tests were used to compare median feeding problems across the different groups of sociodemographic variables (Supplementary Table S1). Education level was significantly associated with feeding problems, wherein caregivers with a postgraduate degree had a significantly higher median score compared to those with lower education levels.

In the multiple linear regression analysis of feeding problems with sociodemographic variables, adjusted for disability category, a positive association was observed between education level and feeding problems (Beta [B] = 1.81, Standard error [SE] = 0.62, 95% Confidence Interval [CI]: 0.58 to 3.04, *p* = 0.004), wherein the education level explained 5% of the change occurring in feeding problems. Similarly, a positive association was found between family monthly income and feeding problems (B = 1.04, SE = 0.45, 95% CI: 0.15 to 1.93, *p* = 0.023), accounting for 4% of the variance. The caregiver’s relationship to the child was also significantly associated with feeding problems (B = 2.79, SE = 0.94, 95% CI: 0.93 to 4.65, *p* = 0.004). When the primary caregiver was the mother, children had lower feeding problem scores. The model explained 6% of the variance in feeding problem. Furthermore, the child’s living arrangement showed a significant positive association with feeding problems (B = 2.37, SE = 0.90, 95% CI: 0.59–4.15, *p* = 0.009), accounting for 5% of the variance (Table 4).

### 3.5. Association Between Feeding Problems and Health-Related Characteristics of the Study Sample

No association was observed between the health-related characteristics of the study sample and feeding issues concerning other medical conditions, untreated dental problems, medication usage, type of medication utilized, structured physical activity, independence level, or sleeping time. The Kruskal–Wallis test indicated similar feeding problem scores across the three different categories of disability (ASD/ADHD/learning disabilities/intellectual disability/other (median 4.00 [1.00–10.00]), cerebral palsy (median 4.50 [0.75–8.00]), and Down syndrome (median 5.0 [0.00–12.5], *p* = 0.899). Data concerning the association between feeding problems and health-related characteristics among the study sample are presented in Supplementary Table S2.

### 3.6. Association Between Feeding Problems and Diet of Children with Developmental Disabilities

Results of the multiple linear regression analysis for the association between feeding problems (independent variable) and diet (dependent variable) in children with DD, adjusted for children’s age, gender, and disability category are presented in Table 5. No link was found between feeding problems and macro- and micronutrient intake, water intake, or diet quality.

## 4. Discussion

The findings of the current study revealed that feeding problems were not prevalent among children with disabilities in this cohort. The most commonly reported issues were related to feeding skills and food selectivity. Specifically, frequent challenges included the inability to self-feed independently, overeating, and pica-like behavior. A significant association was found between the education level of caregivers and feeding problems, with children of caregivers holding postgraduate degrees exhibiting significantly higher feeding problems compared to other groups.

Results reported in the present study indicated that the inability to self-feed independently was the most frequently reported feeding problem among the sampled children. This is consistent with previous research showing that children with neurological impairments, such as cerebral palsy, often experience deficits in fine motor coordination and oral-motor skills, resulting in prolonged dependency on caregivers during mealtimes [27,28]. Children with ADHD may also face challenges with independent self-feeding. While they typically possess the physical ability to feed themselves, difficulties are often related to behavioral dysregulation and inattention sensitivities, which interfere with mealtime routines and sustained focus on eating [29]. Similarly, children with ASD may also struggle with self-feeding, primarily due to sensory processing difficulties, such as aversions to specific tastes, textures, smells, or food appearances [30]. A narrative review found one in five children with ASD displayed significant sensory-based food selectivity, frequently refusing items due to sensory characteristics [31]. Given that most children in our sample were diagnosed with cerebral palsy (22%), ADHD (35%), and ASD (44%), this likely explains the high incidence of self-feeding difficulties observed in the study. A study by Goday et al. (2019) emphasized that self-feeding challenges in this population are associated with reduced dietary variety and lower caloric intake, placing affected children at greater risk of malnutrition [32]. Therefore, early intervention programs focusing on feeding skill development, occupational therapy, and caregiver education to promote greater independence are essential to improve nutritional outcomes in children with disabilities [27].

This study also found that a considerable percentage of children with disabilities expressed overeating behavior and continuing eating as long as food was available. This pattern of dysregulated appetite control is consistent with previous research suggesting that children with developmental or intellectual disabilities may experience impaired satiety signaling and diminished self-regulation, leading to overeating behavior. Nadeau et al. documented a similar pattern in children with ASD, who were observed to consume food even in the absence of hunger cues [33]. Additionally, another study reported a significant positive association between emotional overeating and symptoms of ADHD among preschool-aged children [34]. Another contributing factor to overeating behavior may be environmental influences, such as the absence of structured mealtime routines, screen time, and inconsistent caregiver responses to food-seeking behavior, all of which can exacerbate these tendencies [35]. Overeating behavior is considered a significant concern, as it may increase the risk of obesity in this population. Evidence from previous studies suggests that overweight and obesity are prevalent issues among children with disabilities, including ADHD and ASD [35,36].

Pica-like behavior was also prevalent among the children with disabilities in our sample. It was previously indicated that pica is a common co-occurrence with autism and developmental disabilities [37]. Fields et al. reported that the prevalence of pica was 23.2% among children with ASD and 8.4% among those with other developmental disabilities [38]. Despite these observations, the associations between pica and specific developmental disorders remain poorly understood. Several contributing factors have been proposed to explain the high prevalence of pica in this population, including sensory processing difficulties and environmental influences such as stressful life events, parental neglect, low caregiver involvement, and a history of child abuse [37,38].

In the current study, children of caregivers with higher educational attainment and family income exhibited significantly more feeding problems compared to those whose caregivers had lower levels of education and income. One possible explanation is that more educated caregivers and those with more financial resources may hold higher expectations regarding their child’s eating behaviors, which could lead to increased reporting of issues such as food selectivity, mealtime refusal, or prolonged feeding durations. Additionally, their greater awareness of clinical feeding difficulties may make them more likely to identify and report even mild or early-stage problems. While this finding may seem unexpected, it is consistent with previous studies that have shown maternal education to be a significant factor influencing children’s dietary patterns. These studies suggest that caregivers with higher education levels are generally more attuned to nutrition-related concerns, which may affect both how they perceive their child’s feeding behaviors and how actively they report them [39,40,41]. This highlights the potential value of structured training programs for caregivers that aim to improve feeding practices and support caregivers regardless of their educational levels. Such programs could include culturally appropriate educational materials and practical guidance on oral-motor skills, management of food selectivity, and behavioral feeding techniques.

Furthermore, the caregiver’s relationship with the child and the child’s living arrangement appeared to be significantly associated with feeding problems. The present study suggests that as the responsibility of caregiving is shifted from mother to fathers, siblings, or others, there is an increased risk of feeding problems. Perhaps children feel more comfortable with maternal feeding or mothers are more experienced to handle feeding challenges with their children compared to siblings or other individuals. Similarly, as the living arrangement is shifted from living with both parents to one parent, another family member, or living in a disability center, there is a greater likelihood for children to experience feeding problems. This could be partially explained by the influence of the family/household composition on child feeding or eating behaviors [42]. For instance, a study suggested that eating together as a family could promote healthy eating by fostering parent–child interaction during family meals and providing an opportunity to establish family food rules within a supportive eating environment [43]. Nevertheless, further studies are warranted to investigate the mechanisms of these associations among children with DD.

This study found no link between feeding problem scores and children’s water intake. Despite the lack of association, children with feeding problems such as food refusal or limited oral-motor skills may struggle with fluid intake for several reasons. For example, children who exhibit dysphagia may avoid liquids due to discomfort or difficulty in swallowing [44]. We also hypothesize that children with disabilities who exhibit high levels of food selectivity may have strong preferences or aversions to specific textures and tastes, making plain water less acceptable compared to flavored beverages or thickened fluids. Therefore, monitoring and actively encouraging adequate water intake remains important for supporting optimal health outcomes among children with DD.

This is the first study to explore feeding problems and their associated factors among children with developmental disabilities in Saudi Arabia. However, the generalizability of the findings may be limited by the income and educational characteristics of the study sample relative to the broader Saudi population. In addition, the limited response rate may influence the representativeness of the sample and introduce potential response bias. Clinical consequences of feeding problems were not assessed. Future longitudinal studies should examine the nutritional and clinical impacts of feeding problems in this population.

## 5. Conclusions

The current study highlighted a range of feeding problems among children with DD. These issues may contribute to both undernutrition due to the inability to self-feed independently and obesity, often linked to overeating behavior and poor appetite regulation. The observed prevalence of pica-like behavior adds another layer of concern, given its potential health risks and its strong association with sensory processing difficulties and psychosocial stressors. Given the high prevalence of overeating, pica-like behavior, and food refusal observed among children with DD, multidisciplinary interventions involving nutritionists, speech and language therapists, occupational therapists, and psychologists may be beneficial to support individualized feeding management. National-level guidelines for routine screening of feeding problems in children with DD are recommended, with an emphasis on early identification of chewing and swallowing difficulties to reduce feeding-related complications such as aspiration risk. Tailored training programs for caregivers that aim to improve feeding practices and support caregivers despite their educational level are encouraged. Future longitudinal research should focus on the long-term nutritional consequences related to feeding problems among children with DD.

## Figures and Tables

**Table 1:** Demographic characteristics of the study sample (*n* = 160).

Variable	*n*	%
Caregiver’s relationship with child		
Mother	139	86.9
Father	14	8.80
Sibling	3	1.90
Others	4	2.50
Caregiver’s age group		
20–30 years	31	19.4
31–40 years	76	47.5
>40 years	53	33.1
Caregiver’s education level		
<High school	45	28.1
High school/Diploma	41	25.6
Bachelor	67	41.9
Postgraduate degree	7	4.40
Caregiver’s marital status		
Married	131	81.9
Single	29	18.1
Family monthly income ^1^		
<SAR 4000	41	25.6
SAR 4000–6000	46	28.8
SAR 6001–10,000	34	21.3
SAR 10,001–15,000	27	16.9
>SAR 15,000	12	7.50
Gender		
Boy	92	57.5
Girl	68	42.5
Age group		
1–3 years	9	5.60
4–8 years	73	45.6
9–13 years	56	35.0
14–18 years	22	13.8
Nationality		
Saudi	143	89.4
Non-Saudi	17	10.6
Order of child		
Only child	18	11.3
Oldest child	37	23.1
Middle child	42	26.3
Youngest child	63	39.4
Living arrangement		
Live with both parents	133	83.1
Live with mother	19	11.9
Live with father	4	2.50
Live with other family member or live in disability center	4	2.50

^1^ SAR: Saudi Riyal. $1 = SAR 3.75.

**Table 2:** Health-related characteristics of the study sample (*n* = 160).

Variable	*n*	%
Type of disability		
ADHD	35	21.9
ASD	44	27.5
Cerebral palsy	22	13.8
Intellectual disability	21	13.1
Learning disability	25	15.6
Down syndrome	10	6.30
Other	3	1.90
Other medical conditions		
No	115	71.9
Yes	45	28.1
Untreated dental problem		
No	90	56.3
Yes	70	43.8
Medication use		
No	129	80.6
Yes	31	19.4
Structured physical activity		
No	119	74.4
Yes	41	25.6
Independency		
No	88	55.0
Yes	72	45.0
Sleep time		
Adequate sleep	61	38.1
Inadequate sleep	99	61.9
Screen time		
≤2 h/d	102	63.8
>2 h/d	58	36.3
Food allergies		
No	146	91.3
Yes	14	8.80
Dietitian visit		
No	123	76.9
Yes	37	23.1
Supplement use		
No	113	70.6
Yes	47	29.4
Nutritional drink use		
No	144	90.0
Yes	16	10.0

**Table 3:** Distribution of responses to the screening tool of feeding problems (STEP-AR) (*n* = 160).

Subdomain		Never	Yes, Occurring Between 1 and 10 Times in the Last Month			Yes, More Than 10 Times in the Last Month			Incidence
			Causes No Problems	Causes Minimal Harm	Causes Serious Harm	Causes No Problems	Causes Minimal Harm	Causes Serious Harm	
Aspiration Risk	He/she regurgitates and re-swallows food either during or immediately following meals	137 (85.6)	14 (8.75)	4 (2.50)	1 (0.63)	3 (1.88)	1 (0.63)	0 (0.00)	14.3%
	He/she vomits either during or immediately following meals	148 (92.5)	5 (3.13)	4 (2.50)	1 (0.63)	1 (0.63)	1 (0.63)	0 (0.00)	7.5%
Refusal	Problem behaviors (e.g., aggression, SIB) increase during mealtimes	141 (88.1)	10 (6.25)	6 (3.75)	2 (1.25)	0 (0.00)	1 (0.63)	0 (0.00)	11.8%
	He/she spits out their food before swallowing	137 (85.6)	13 (8.13)	5 (3.13)	1 (0.63)	3 (1.88)	1 (0.063)	0 (0.00)	14.3%
	He/she pushes food away or attempts to leave the area when food is presented	116 (72.5)	23 (14.4)	11 (6.88)	1 (0.63)	3 (1.88)	5 (3.13)	1 (0.63)	27.5%
Selectivity	He/she will only eat selected types of food (e.g., pudding, rice)	117 (73.1)	27 (16.9)	4 (2.50)	1 (0.63)	8 (5.00)	2 (1.25)	1 (0.63)	26.8%
	He/she prefers a certain setting for eating (e.g., bedroom, dining room)	133 (83.1)	13 (8.13)	2 (1.25)	1 (0.63)	9 (5.63)	2 (1.25)	0 (0.00)	16.8%
	He/she prefers to be fed by a specific caregiver or prefers to be fed rather than feed himself/herself	111 (69.4)	33 (20.6)	5 (3.13)	0 (0.00)	4 (2.50)	5 (3.13)	2 (1.25)	30.6%
	He/she only eats foods of certain textures	114 (71.3)	25 (15.6)	8 (5.00)	1 (0.63)	8 (5.00)	3 (1.88)	1 (0.63)	28.7%
Nutrition Behaviors	He/she eats or attempts to eat items that are not food	110 (68.8)	29 (18.1)	9 (5.63)	2 (1.25)	5 (3.13)	4 (2.50)	1 (0.63)	31.2%
	He/she only eats a small amount of the food presented to him or her	119 (74.3)	22 (13.7)	4 (2.50)	1 (0.63)	6 (3.75)	7 (4.38)	1 (0.63)	25.6%
	He/she will continue to eat as long as food is available	107 (66.8)	33 (20.6)	10 (6.25)	2 (1.25)	5 (3.13)	2 (1.25)	1 (0.63)	33.1%
Skills	He/she cannot feed him/herself independently	97 (60.6)	35 (21.8)	13 (8.13)	5 (3.13)	9 (5.63)	1 (0.63)	0 (0.00)	39.3%
	He/she does not demonstrate the ability to chew	114 (71.2)	27 (16.8)	9 (5.63)	1 (0.63)	6 (3.75)	3 (1.88)	0 (0.00)	28.7%
	He/she chokes on food	138 (86.2)	11 (6.88)	4 (2.50)	3 (1.88)	2 (1.25)	2 (1.25)	0 (0.00)	13.7%
	He/she does not demonstrate the ability to swallow	126 (78.7)	17 (10.6)	6 (3.75)	2 (1.25)	8 (5.00)	1 (0.63)	0 (0.00)	21.2%
	He/she eats a large amount of food in a short period of time	122 (76.2)	21 (13.1)	7 (4.38)	1 (0.63)	5 (3.13)	3 (1.88)	1 (0.63)	23.7%

Numbers presented in table are frequencies and percentages.

**Table 4:** Multiple linear regression analysis of feeding problems with sociodemographic characteristics of the sample.

Variable	Beta	Standard Error	*p*-Value	95% Confidence Interval	R-Squared
Caregiver’s relationship with child (Mother = 1; Father = 2; Sibling = 3; Others = 4)	2.79	0.94	0.004 *	0.93 to 4.65	0.06
Caregiver’s age group (20–30 years = 1; 31–40 years = 2; >40 years = 3)	−0.69	0.81	0.399	−2.29 to 0.92	0.01
Caregiver’s education level (<High school = 1; High school/diploma = 2; Bachelor = 3; Postgraduate degree = 4)	1.81	0.62	0.004 *	0.58 to 3.04	0.05
Caregiver’s marital status (Married = 1; Single = 2)	0.99	1.49	0.507	−1.95 to 3.93	0.01
Family monthly income (<SAR 4000 = 1; SAR 4000–6000 = 2; SAR 6001–10,000 = 3; SAR 10,001–15,000 = 4; >SAR 15,000 = 5)	1.04	0.45	0.023 *	0.15 to 1.93	0.04
Other children with disability (No = 0; Yes = 1)	0.36	1.63	0.824	−2.86 to 3.58	0.00
Children’s gender (Boy = 1; Girl = 2)	0.57	1.17	0.625	−1.74 to 2.88	0.00
Children’s age group (1–3 years = 1; 4–8 years = 2; 9–13 years = 3; 14–18 years = 4)	0.06	0.72	0.937	−1.37 to 1.49	0.00
Nationality	−0.25	1.91	0.895	−4.03 to 3.52	0.00
Order of child (Only child = 1; Oldest child = 2; Middle child = 3; Youngest child = 4)	−0.73	0.55	0.189	−1.82 to 0.36	0.01
Living arrangement (Live with both parents = 1; Live with mother = 2; Live with father = 3; Live with other family member or in disability center = 4)	2.37	0.90	0.009 *	0.59 to 4.15	0.05

* Statistically significant with alpha = 0.05. All models were adjusted for disability category.

**Table 5:** Multiple linear regression analysis of feeding problems with dietary intake.

Variable	Beta	Standard Error	*p*-Value	95% Confidence Interval	R-Squared
Diet quality score	0.01	0.02	0.622	−0.03 to 0.05	0.01
Water, cup/d	−0.05	0.02	0.051	−0.09 to −0.00	0.12
Energy, kcal/d	2.33	20.5	0.911	−39.9 to 44.6	0.08
Carbohydrate, g/d	−1.38	3.06	0.656	−7.69 to 4.93	0.06
Protein, g/d	0.54	1.86	0.775	−3.28 to 4.35	0.15
Fat, g/d	0.28	0.93	0.763	−1.62 to 2.18	0.12
Fiber, g/d	0.29	0.56	0.614	−0.86 to 1.43	0.16
Free sugar, g/d	1.18	2.26	0.606	−3.47 to 5.83	0.31
Sodium	−286	160	0.085	−614 to 42.5	0.17
Potassium	−0.94	37.8	0.980	−78.7 to 76.8	0.00
Calcium	−1.12	12.3	0.928	26.4 to 24.2	0.04
Phosphorus	−6.40	12.80	0.621	−32.71 to 19.91	0.04
Magnesium	−1.11	2.46	0.657	−6.17 to 3.96	0.20
Iron	−0.01	0.20	0.970	−0.42 to 0.40	0.02
Zinc	−0.06	0.10	0.586	−0.26 to 0.15	0.08
Copper	−0.29	0.55	0.602	−1.43 to 0.84	0.07
Manganese	0.29	2.90	0.920	−5.67 to 6.26	0.16
Selenium	−0.07	1.29	0.955	−2.72 to 2.57	0.06
Iodine	−0.80	1.22	0.516	−3.31 to 1.70	0.08
Vitamin D	0.60	1.50	0.694	−2.50 to 3.69	0.09
Vitamin E	−0.04	0.13	0.784	−0.30 to 0.23	0.05
Vitamin B12	−0.003	0.19	0.988	−0.39 to 0.39	0.07
Vitamin C	0.03	1.55	0.987	−3.15 to 3.21	0.06

Significant at 95% confidence level. All models were adjusted for child age, gender, and disability category. Data for diet quality score were available for 151 children, while data for dietary intake were available for 32 children.

## Data Availability

The data presented in this study are available upon request from the corresponding author due to privacy and ethical purposes.

## Supplementary Materials

The following supporting information can be downloaded at: https://www.mdpi.com/article/10.3390/nu18020356/s1.

## Abbreviations

The following abbreviations are used in this manuscript:

DD	Developmental Disabilities
STEP-AR	Validated Screening Tool for Eating/Feeding Problems
ADHD	Attention Deficit Hyperactivity Disorder
ASD	Autism Spectrum Disorder
SFFFQ	Short-Form Food Frequency Questionnaire
