# Supplementary material for: Investigation of Feeding Problems and Their Associated Factors in Children with Developmental Disabilities in Saudi Arabia
Source: Nutrients. 2026 Jan 22;18(2):356. doi: 10.3390/nu18020356 (PMC12844803; doi:10.3390/nu18020356)
Supplement: Supplementary file 1 [file nutrients-18-00356-s001.zip › Supplementary Materials S1.pdf]

Table S1: Bivariate associations between feeding problem and sociodemographic characteristics of the sample (n = 160).

Variable	Mean ± SD	Median (IQR)	*p-value*
**Caregiver's relationship with child**			
Mother	5.91 ± 5.92	4.00 (1.00-9.00)	0.437
Father	5.21 ± 6.20	3.50 (0.00-9.25)	
Sibling	9.00 ± 13.9	2.00 (0.00-NA)	
Others	19.0 ± 22.7	12.0 (2.75-42.2)	
**Caregiver's age group**			
20-30 years	7.06 ± 5.77	7.00 (2.00-10.0)	0.292
31-40 years	6.30 ± 6.98	4.00 (0.00-9.00)	
>40 years	5.64 ± 7.94	4.00 (0.50-10.0)	
**Caregiver's education level**			
< High school	3.98 ± 4.68	3.00 (0.00-6.00)	0.009*
High school/Diploma	6.39 ± 8.75	5.00 (1.50-8.00)	
Bachelor	6.94 ± 6.45	5.00 (2.00-11.0)	
Postgraduate degree	13.0 ± 10.2	10.0 (7.00-23.0)	
**Caregiver's marital status**			
Married	6.04 ± 6.04	5.00 (2.00-9.00)	0.612
Single	7.10 ± 10.7	4.00 (0.00-10.5)	
**Family monthly income**			
SAR < 4,000	4.66 ± 5.68	3.00 (0.00-8.50)	0.055
SAR 4,000-6,000	5.57 ± 8.27	4.00 (0.00-8.00)	
SAR 6,000-10,000	7.15 ± 7.01	5.50 (1.75-10.0)	
SAR 10,000-15,000	7.40 ± 7.10	5.00 (3.00-11.0)	
SAR > 15,000	7.81 ± 7.17	5.00 (3.00-11.0)	
**Other children with disability**			
No	6.18 ± 7.30	4.00 (1.00-9.00)	0.414
Yes	6.60 ± 5.77	5.00 (3.00-10.0)	
**Gender**			
Boy	5.93 ± 7.10	4.00 (1.00-9.00)	0.624
Girl	6.63 ± 7.09	4.50 (1.00-10.0)	
**Age group**			
1-3 years	5.78 ± 4.66	6.00 (1.00-9.00)	0.557
4-8 years	6.60 ± 6.12	5.00 (1.50-10.0)	
9-13 years	5.41 ± 7.48	4.00 (0.00-8.75)	
14-18 years	7.27 ± 9.65	3.00 (0.00-11.5)	
**Nationality**			
Saudi	6.22 ± 7.27	4.00 (1.00- 10.0)	0.600
Non-Saudi	6.29 ± 5.46	6.00 (1.50-9.00)	
**Order of child**			

Only child	10.2 ± 11.6	10.0 (3.50-12.2)	0.108
Oldest child	5.78 ± 5.49	4.00 (2.00-9.00)	
Middle child	5.00 ± 5.99	4.00 (0.00-7.25)	
Youngest child	6.17 ± 6.70	5.00 (0.00-9.00)	
**Living arrangement**			
Live with both parents	5.99 ± 6.05	4.00 (1.50-9.00)	0.263
Live with mother	5.32 ± 5.39	4.00 (0.00-10.0)	
Live with father	3.00 ± 3.83	2.00 (0.00-7.00)	
Live with other family member or live in disability center	22.0 ± 22.5	18.0 (2.75-45.2)	

* Significant at 95% confidence level. *p*-value presented in this table was obtained using Mann-Whitney U and the Kruskal-Wallis tests.
