# Supplementary material for: Investigation of Feeding Problems and Their Associated Factors in Children with Developmental Disabilities in Saudi Arabia
Source: Nutrients. 2026 Jan 22;18(2):356. doi: 10.3390/nu18020356 (PMC12844803; doi:10.3390/nu18020356)
Supplement: Supplementary file 1 [file nutrients-18-00356-s001.zip › Supplementary Materials S2.pdf]

Table S2: Association between feeding problem and health-related characteristics of the sample (n = 160).

	Mean ± SD	Median (IQR)	*p-value*
**Type of disability**			
ADHD/ASD, LD, ID, Other	6.38 ± 7.46	4.00 (1.00-10.0)	0.899
Cerebral Palsy	5.32 ± 5.21	4.50 (0.75-8.00)	
Down Syndrome	6.40 ± 5.93	5.00 (0.00-12.50)	
**Other medical conditions**			
No	5.53 ± 5.86	4.00 (0.00-9.00)	0.107
Yes	8.02 ± 9.37	5.00 (2.00-11.50)	
**Untreated dental problem**			
No	5.35 ± 5.52	4.00 (0.00-9.00)	0.122
Yes	7.39 ± 8.52	5.50 (2.00-10.0)	
**Medication use**			
No	6.06 ± 6.35	4.00 (1.00-9.00)	0.848
Yes	6.94 ± 9.66	4.00 (2.00-10.0)	
**Structured physical activity**			
No	6.13 ± 7.29	4.00 (1.00-9.00)	0.744
Yes	6.54 ± 6.50	5.00 (0.00-10.0)	
**Independency**			
No	6.77 ± 7.98	4.00 (2.00-10.0)	0.442
Yes	5.57 ± 5.78	4.00 (0.00-9.00)	
**Sleep time**			
Adequate sleep	6.11 ± 5.91	4.00 (1.00-10.0)	0.595
Inadequate sleep	6.30 ± 7.75	4.00 (0.00-9.00)	
**Screen time**			
1-2 h/d	6.38 ± 7.65	4.00 (0.75-9.25)	0.922
> 2 h/d	5.97 ± 6.04	4.50 (1.00-10.0)	
**Food allergies**			
No	6.16 ± 7.31	4.00 (0.75-9.25)	0.152
Yes	7.00 ± 4.04	8.50 (2.75-10.0)	
**Dietitian visit**			
No	6.20 ± 7.22	4.00 (1.00-9.00)	0.858
Yes	6.32 ± 6.70	5.00 (0.00-10.0)	
**Supplement use**			
No	5.87 ± 7.22	4.00 (0.00-9.00)	0.154
Yes	7.11 ± 6.73	5.00 (2.00-11.0)	
**Nutritional drink use**			
No	6.06 ± 7.17	4.00 (1.00-9.00)	0.158
Yes	7.81 ± 6.20	7.00 (3.25-12.25)	

* Significant at 95% confidence level. *p*-value presented in this table was obtained using Fisher's Exact test.
